# Evaluating antitumor activity of *Escherichia coli* purine nucleoside phosphorylase against head and neck patient‐derived xenografts

**DOI:** 10.1002/cnr2.1708

**Published:** 2022-10-17

**Authors:** Regina Rab, Annette Ehrhardt, Bhagelu R. Achyut, Disha Joshi, Melissa Gilbert‐Ross, Chunzi Huang, Katharine Floyd, Anton V. Borovjagin, William B. Parker, Eric J. Sorscher, Jeong S. Hong

**Affiliations:** ^1^ Department of Pediatrics and Children's Hospital of Atlanta Emory University School of Medicine Atlanta Georgia USA; ^2^ Winship Cancer Institute Emory University School of Medicine Atlanta Georgia USA; ^3^ Department of Biomedical Engineering University of Alabama at Birmingham Birmingham Alabama USA; ^4^ Department of Pharmacology University of Alabama at Birmingham; PNP Therapeutics, Inc. Birmingham Alabama USA

**Keywords:** gene transfer, head and neck squamous cell carcinoma, patient‐derived xenografts, purine nucleoside phosphorylase, tropism modified adenovirus

## Abstract

**Background:**

Purine nucleoside phosphorylase (PNP) gene transfer represents a promising approach to treatment of head and neck malignancies. We tested recombinant adenovirus already in phase I/II clinical testing and leading‐edge patient‐derived xenografts (PDX) as a means to optimize this therapeutic strategy.

**Methods:**

Our experiments investigated purine base cytotoxicity, PNP enzyme activity following treatment of malignant tissue, tumor mass regression, viral receptor studies, and transduction by tropism‐modified adenovirus.

**Results:**

Replication deficient vector efficiently transduced PDX cells and mediated significant anticancer effect following treatment with fludarabine phosphate in vivo. Either 6‐methylpurine or 2‐fluoroadenine (toxic molecules generated by the PNP approach) ablated head and neck cancer cell proliferation. High levels of adenovirus‐3 specific receptors were detected in human tumor models, and vector was evaluated that utilizes this pathway.

**Conclusions:**

Our studies provide the scientific foundation necessary to improve PNP prodrug cleavage and advance a new treatment for head and neck cancer.

## 1 INTRODUCTION

Therapeutic gene transfer comprises an important experimental approach to treatment of refractory solid tumors. Such strategies have been tested clinically with encouraging results against head and neck squamous cell carcinoma (HNSCC) using a modified *Escherichia coli* purine nucleoside phosphorylase (PNP) gene and replication deficient adenovirus.^1^, ^2^, ^3^, ^4^, ^5^ PNP converts specific nucleosides to highly active purine base molecules that inhibit refractory tumors. Cancer killing by this mechanism engages a unique mode of action and disrupts DNA, RNA, and protein synthesis—leading to robust preclinical effectiveness against numerous tumor cell types in vitro and in vivo.^5^, ^6^, ^7^, ^8^, ^9^, ^10^, ^11^, ^12^, ^13^, ^14^, ^15^, ^16^, ^17^, ^18^, ^19^ Moreover, PNP is active even when a very small fraction of cancer cells (e.g., <2.5% of a tumor mass) express the transgene.^3^, ^4^, ^7^, ^8^ This level of bystander cell killing is unprecedented, and the approach is fundamentally distinct from earlier interventions using transgenes such as thymidine kinase or cytosine deaminase.^1^, ^2^, ^3^, ^4^, ^5^

Fludarabine phosphate (F‐araAMP), a drug approved for treatment of chronic lymphocytic leukemia but with no activity against epithelial or other solid neoplasms, has been successfully applied to the PNP strategy in vivo. F‐araAMP is rapidly converted in blood to fludarabine (F‐ araA), which is cleaved by *E. coli* PNP to 2‐fluoroadenine (F‐Ade), a purine base capable of abolishing large solid tumors. The PNP/F‐araAMP combination has been shown safe in both animal models and human subjects.^2^, ^3^, ^5^ F‐Ade that escapes cancer parenchyma is markedly diluted in the host and metabolized by a ubiquitous enzyme (xanthine oxidase) to levels non‐toxic (and undetectable) in vivo.^1^, ^2^, ^3^, ^4^, ^5^, ^20^ The approach has elicited tumor regressions during Phase I testing, and is presently undergoing Phase I/II clinical analysis against HNSCC.

Although PNP appears promising as a treatment for solid neoplasms of the head and neck, work remains in order to optimize effectiveness, inhibit heavily pretreated cancer tissues, and adequately transduce tumor parenchyma in human subjects. Preclinical development has been severely impeded by a paucity of well‐characterized head and neck cancer models that can propagate efficiently and recapitulate aspects of the human neoplasm. In addition, essential tools for augmenting bystander cell killing and improving viral uptake are not available. In the present study, we tested leading‐edge head and neck tumors recently made available from the NCI Patient‐Derived Models Repository (PDMR) that faithfully reproduce many aspects of in vivo cancer biology.^21^ Our specific objective was to develop patient‐derived xenograft (PDX) models for studies of HNSCC gene transfer, including experiments to evaluate: (1) malignant cell killing by purine base compounds (6‐methylpurine [MeP], F‐Ade), (2) transduction with recombinant adenovirus encoding *E. coli* PNP, (3) in vivo anticancer activity, (4) protein receptors available in situ for nucleotide delivery to PDX tumors, and (5) viral fiber modification for enhanced transduction efficiency. By applying PNP gene transfer—an approach emblematic of other nucleotide‐based treatment strategies—we demonstrate strong tumor susceptibility to purine cytotoxins, high efficiency adenoviral gene delivery, antitumor responsiveness in vivo, and “proof‐of‐concept” for a retargeted, tropism‐modified viral vector.

## 2 MATERIALS AND METHODS

### 2.1 Well characterized PDX models of HNSCC

Patient‐derived mixed/crude (non‐clonal) tumor cells were obtained according to leading edge NCI PDMR protocols (Frederick, MD; https://pdmr.cancer.gov/) based on banking of cryopreserved patient tumor tissues. Extensive quality control of cancer models from the PDMR evaluates features such as short tandem repeat (STR) profiling, qRT‐PCR‐verified tumor origin, pathology and/or immunohistochemistry of malignant subtype, and concordance between STR profile and whole exome sequencing. Specific cancer models studied here were: 328373‐195‐R‐J1‐PDC (head and neck squamous cell carcinoma from a lateral neck soft tissue mass), as well as 929823‐356‐R‐J2‐PDC; 958767‐090‐R‐J1‐PDC; and 845751‐090‐R‐J2‐PDC (lip/oral cavity squamous cell carcinomas). Tissue was propagated according to NCI guidance (https://pdmr.cancer.gov/sops/default.htm) with 500 ml tumor cell growth media containing: 473 ml advanced DMEM/F12 1X supplemented with 5% FBS, 177 μM adenine, 0.4 μg/mL hydrocortisone, 0.01 μg/mL EGF recombinant human protein, 100 U/mL pen/strep, 2 mM L‐glutamine, and 10 μM Y‐27632 dihydrochloride.

### 2.2 In vitro cell killing with purine base molecules

PDX cells were seeded into 6‐well or 24‐well plates and MeP, F‐Ade, or F‐adenosine added at 10–200 μM 24 h after plating. Cells were monitored for 5 days and stained with crystal violet^22^ to evaluate cell survival.

### 2.3 PNP and other transgene activity

Approximately 50 mg PDX lysate (from in vivo tissue or cell culture) were frozen and stored at −80°C. Crude extracts were prepared^8^ and incubated with 50 mM potassium phosphate together with 100 μM MeP‐dR (*E. coli* PNP substrate) in 100 mM HEPES buffer (pH 7.4) at a concentration of lysate that resulted in a linear signal during the incubation period. Formation of MeP cytotoxin was monitored using reverse phase HPLC (Perkin Elmer). A similar protocol was used to test PDX extracts from PNP‐transduced tumors at 72 h following gene transfer. In other expression studies, luciferase activity was measured using the Bright‐Glo reagent (Promega) according to manufacturer's instructions on a Flex Station luminometer (Molecular Devices).

### 2.4 In vivo establishment of PDX tumors

328373‐195‐R‐J1‐PDC cells were cultured on 15 cm plates, harvested by trypsinization, and resuspended in PBS (1 × 10^8^ cells/ml). NOD‐scid‐gamma (NSG) (female, aged 6–8 weeks) mice were inoculated with 1 × 10^7^ 328373‐195‐R‐J1‐PDC cells subcutaneously (150 μl/injection) in each flank. Note that tumor mass in studies such as these can be estimated by a conventional caliper measurement using the equation: (length x width squared)/2 = cubic mm and converted to mg assuming unit density.^1^ (By this method, a calculated tumor volume of 100 mm^3^ represents mass of approximately 100 mg.) Tumors were treated when they reached a size of 100–300 mm^3^. Tumor‐bearing mice were randomly divided into two experimental groups as shown in Table 1. HNSCC xenografts were useful for single‐cycle intratumoral dosing of adenovirus, and exhibit suitable doubling time so that antitumor activity can be assessed. Multiple cycles of therapy and parenchymal damage from repetitive needle inoculations (as well as repeat chemotherapy administration) can be more difficult to evaluate in this setting.

**TABLE 1:** In vivo tumor schedule

Days after PDX cell injection	Days 32 and 33	Days 36, 37, 38	Days 43, 44	Day 47
Group 1	Ad5‐PNP injection^a^	Fludarabine phosphate^b^	Ad5‐PNP injection	Tumor harvested for PNP activity measurements
Group 2 (control)	Sterile PBS injection^a^	Fludarabine phosphate^b^	Sterile PBS injection	

Abbreviations: Ad5‐PNP, adenovirus serotype‐5 encoding purine nucleoside phosphorylase; PBS, phosphate buffered saline; PDX, patient‐derived xenograft.

^a^ Tumors reached approximate size for injection (100–300 mm^3^ Section 2) by Day 32. Each tumor was dosed with eight needle inoculations/treatment, three times per day for 2 days. Injections contained approximately 20 μl of viral inoculum from a total of 150 μl (2 × 10^11^ virus particles) or sterile PBS (phosphate buffered saline).

^b^ To allow for optimal PNP gene expression, at 72 h following viral transduction, 167 mg/kg fludarabine phosphate was administered intraperitoneally, three times/day at 4‐h intervals for 3 days. Each injection was set at 250 μl/25 g body weight.

### 2.5 Adenoviral transduction of PDX models

Recombinant adenovirus was administered to HEK293 or PDX lines and intracellular viral genomic DNA evaluated by copy number variance ddPCR. Briefly, 4 × 10^12^ viral particles were added to PDX cells in Opti‐MEM. Following 4‐h incubation to allow entry, cells were collected by trypsinization and washed with PBS to remove surface bound viral DNA/particles. Genomic DNA (viral and cellular) was prepared using the DNeasy Blood and Tissue kit (Qiagen). Copy number variance was determined with a QX200 ddPCR system (Bio‐Rad) according to manufacturer's protocol (https://www.bio-rad.com/webroot/web/pdf/lsr/literature/10033173.pdf). Probes for the assay are described in Table 2. Luciferase expression was quantified as above for 1 × 10^5^ PDX cells infected with 4 × 10^8^ viral particles at 48 h post‐infection.

**TABLE 2:** Gene assays

Target gene	Assay type	Assay ID/catalog #	Probe fluorophore	Manufacturer
CXADR (Ad5 receptor)	Gene expression	Hs03851234_s1	FAM	Applied Biosystems
CD46 (Ad3 receptor)	Gene expression	Hs00611257_m1	FAM	Applied Biosystems
DSG2 (Ad3 receptor)	Gene expression	dHsaCPE5053002	FAM	Bio‐Rad
ATP5F1^31^ (reference gene)	Gene expression	dHsaCPE5035189	HEX	Bio‐Rad
Human adenovirus 5, Hexon (L3)^a^	Pathogen	Pa03453412_s1	FAM	Applied Biosystems
TERT^a^	Copy number variation	4403316	VIC	Applied Biosystems

Abbreviations: ATP5F1, adenosine triphosphate synthase F1; CD46, high affinity surface receptor for adenovirus serotype 3; CXADR, coxsackievirus and adenovirus receptor; DSG2, desmoglein 2; FAM, fluorescein amidite; HEX, hexachlorofluorescein for labeling oligonucleotides; TERT, telomerase reverse transcriptase; VIC, fluorescein dye for labeling oligonucleotides.

^a^ For counting viral particles per cell, Hexon is ratioed to TERT.

### 2.6 mRNA abundance of adenoviral surface receptors in PDX tumor models

mRNA copy number for specific adenoviral receptors was measured by ddPCR using the Bio‐Rad QX200 autoDG ddPCR system. Total RNA was purified with an RNeasy mini kit (Qiagen), and cDNA synthesized using SuperScript IV VILO master mix (ThermoFisher, MA, USA). A cDNA aliquot equivalent to 10 ng total RNA was applied to each reaction for measurement of mRNA. ddPCR was performed according to manufacturer's instructions (https://www.bio-rad.com/webroot/web/pdf/lsr/literature/10048730.pdf). Target gene expression was multiplexed using HEX labeled ATP5F1 gene probes (as a reference to minimize variability) and other probes as summarized in Table 2.

### 2.7 Transduction by retargeted (tropism‐modified) adenovirus

Construction of Ad5‐PNP has been described previously.^7^ Transgene expression cassettes were used to replace a deleted E1 region and incorporate PNP or luciferase under control of the CMV early promoter. Ad5‐wt luc is a luciferase‐expressing recombinant Ad5 (subgroup C) with an unmodified fiber receptor binding domain. Ad5‐F5/3‐luc recombinant virus encodes a receptor binding (C‐terminal) domain of Ad5 fiber genetically replaced with the corresponding domain of Ad3 (subgroup B) to allow binding with Ad3/subgroup B‐specific cell surface receptors.^23^

## 3 RESULTS

### 3.1 In vitro purine base tumor cell killing

**FIGURE 1:**
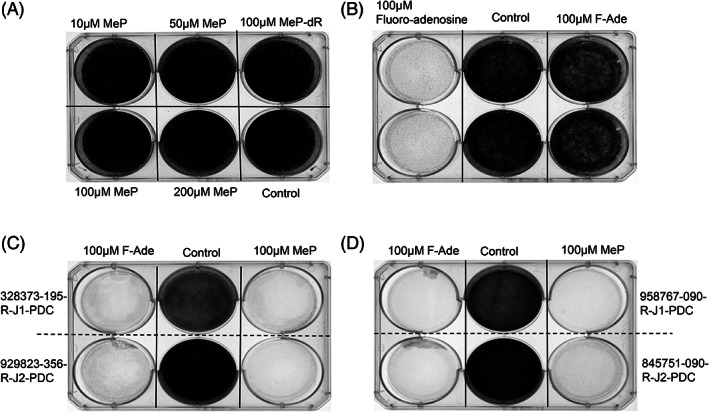
Purine base toxins (MeP or F‐Ade) ablate cancer cell growth. Four human head and neck tumor lines (Section 2) were tested for cell killing by purine base administration. After plating 5 × 10^4^ (A) or 5 × 10^5^ (B) cells/well, drug treatment was administered for 5 days at concentrations shown. Cell staining with 0.1% crystal violet was used to track viability. (A,B) Tumor cell killing in an HNSCC PDX line (929823‐356‐R‐J2‐PDC) was resistant when cultured in standard NCI media despite high concentrations of MeP or F‐Ade (10–200 μM). Results are representative of four PDX tumor cell lines with similar findings. Clear wells indicate >95% cell killing. (B) Fluoro‐adenosine (activated by adenosine kinase to F‐AMP) abolished tumor cell growth, suggesting the presence of an inhibitor in culture medium that completely blocked F‐Ade cellular entry or metabolism, leading to failure of F‐Ade activation to F‐AMP. (C,D) Four human tumor models were efficiently destroyed by incubating with F‐Ade or MeP when cells were cultured using media lacking excess adenine

Highly toxic purine molecules liberated by *E. coli* PNP are effective mediators of solid tumor regression in numerous preclinical settings.^5^, ^6^, ^7^, ^8^, ^9^, ^10^, ^11^, ^12^, ^13^, ^14^, ^15^, ^16^, ^17^, ^18^, ^19^ To investigate whether purines such as MeP or F‐Ade (toxins elaborated by PNP from the nucleosides 6‐methylpurine 2′‐deoxyriboside [MeP‐dR] and fludarabine, respectively) would inhibit head and neck PDX cells, we conducted initial experiments in vitro. Addition of MeP or F‐Ade—which kill by interrupting DNA, RNA, and protein synthesis—failed to inhibit cell growth under standard NCI culture conditions (Figure 1A). 2‐Fluoro‐adenosine (a cytotoxic nucleoside metabolized to the same active intermediates as F‐Ade) was also tested to rule out more generalized resistance against purine molecules by PDX lines (Figure 1B). Interestingly, 2‐fluoro‐adenosine exhibited strong tumor cell inhibition, suggesting that NCI‐recommended growth media contains a component that blocks activity of MeP or F‐Ade. Since all three compounds ablate tumor cells through the same downstream pathways, the observation that fluoroadenine potently destroys HNSCC in vitro specifically when adenine is omitted from growth medium (compare Figures 1B versus C, D) suggests competitive inhibition of MeP and F‐Ade by adenine. The NCI media (Section 2) includes adenine supplementation (177 μM). When cell culture conditions omitted excess adenine, HNSCC cells were completely abolished by MeP or F‐Ade in all four PDX models tested (Figure 1C,D).

### 3.2 Measurement of PNP activity following transduction by Ad5‐PNP

**FIGURE 2:**
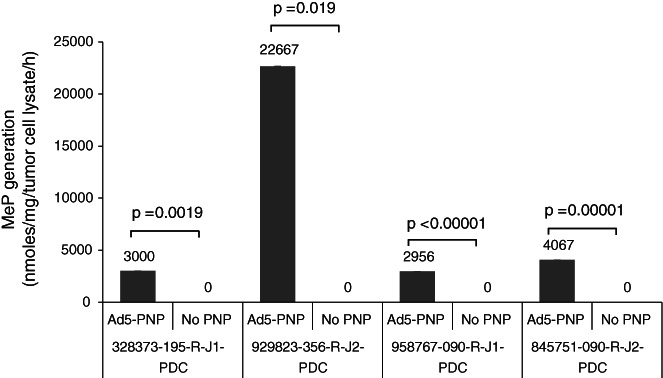
Ad5‐PNP gene transfer in human head and neck cancer lines results in excellent transgene activity. Human PDX tumor cells were grown in 24‐well plates and either treated with vehicle or replication‐deficient Ad5 encoding PNP. Ad5‐PNP was dosed at 4 × 10^12^ viral particles/well with MeP measurement at 72 h post‐transduction. *p* values calculated by student's *t* test

Because PNP activity levels represent a useful correlative endpoint for predicting tumor regressions in vivo,^3^, ^5^, ^7^ we determined the extent to which Ad5‐PNP mediates expression of functional PNP enzyme. Ad5‐PNP‐treated HNSCC cells were sonicated at 3 days post infection and lysates assayed using reverse phase HPLC. Cancer cells transduced by Ad5‐PNP exhibited high‐level PNP activity, which varied depending on the PDX model tested (Figure 2). PNP levels were determined to be at or above the magnitude shown previously to elicit tumor regression in combination with systemic MeP‐dR or F‐araAMP in vivo.^6^, ^7^, ^8^, ^24^, ^25^

### 3.3 Intratumoral transduction of head and neck tumors with Ad5‐PNP

A clinical trial currently in progress employs intratumoral adenovirus encoding a C‐terminal modified *E. coli* PNP to treat needle accessible HNSCC.^20^ As described above, bystander killing by PNP differs fundamentally from first generation systems using enzymes such as thymidine kinase or cytosine deaminase.^1^, ^2^, ^3^, ^4^, ^5^ Due to lack of well‐standardized HNSCC tumor models obtained from human subjects, earlier preclinical evidence in support of the PNP strategy has largely depended on glioma and other non‐HNSCC neoplasms. The need to develop and optimize PNP against human HNSCC, together with importance of advanced tools for improving PNP efficacy, prompted us to test the same Ad5‐PNP being used in human subjects against PDX tumors in mice. We established HNSCC neoplasms by injecting 1 × 10^7^ 328373‐195‐R‐J1‐PDC cells bilaterally into the flanks of NOD scid gamma (NSG) or nude mice. Once tumors reached a size of 100–300 mm^3^, Ad5‐PNP was assessed by dosing 2 × 10^11^ viral particles intratumorally in 150 μl three times a day over 2 days. Seventy‐two hours following viral administration, tumors were harvested and in vitro conversion of MeP‐dR to MeP determined using tissue lysates. We found that transduced human xenografts converted MeP‐dR at rates of 100–1000 nmoles per mg tumor tissue per hour, which is in a range demonstrated previously to confer strong in vivo regressions.^3^, ^6^, ^7^, ^8^, ^24^, ^25^ No *E. coli* PNP activity was observed in control (vehicle treated) tumors (not shown).

### 3.4 PNP treatment in combination with F‐araAMP

**FIGURE 3:**
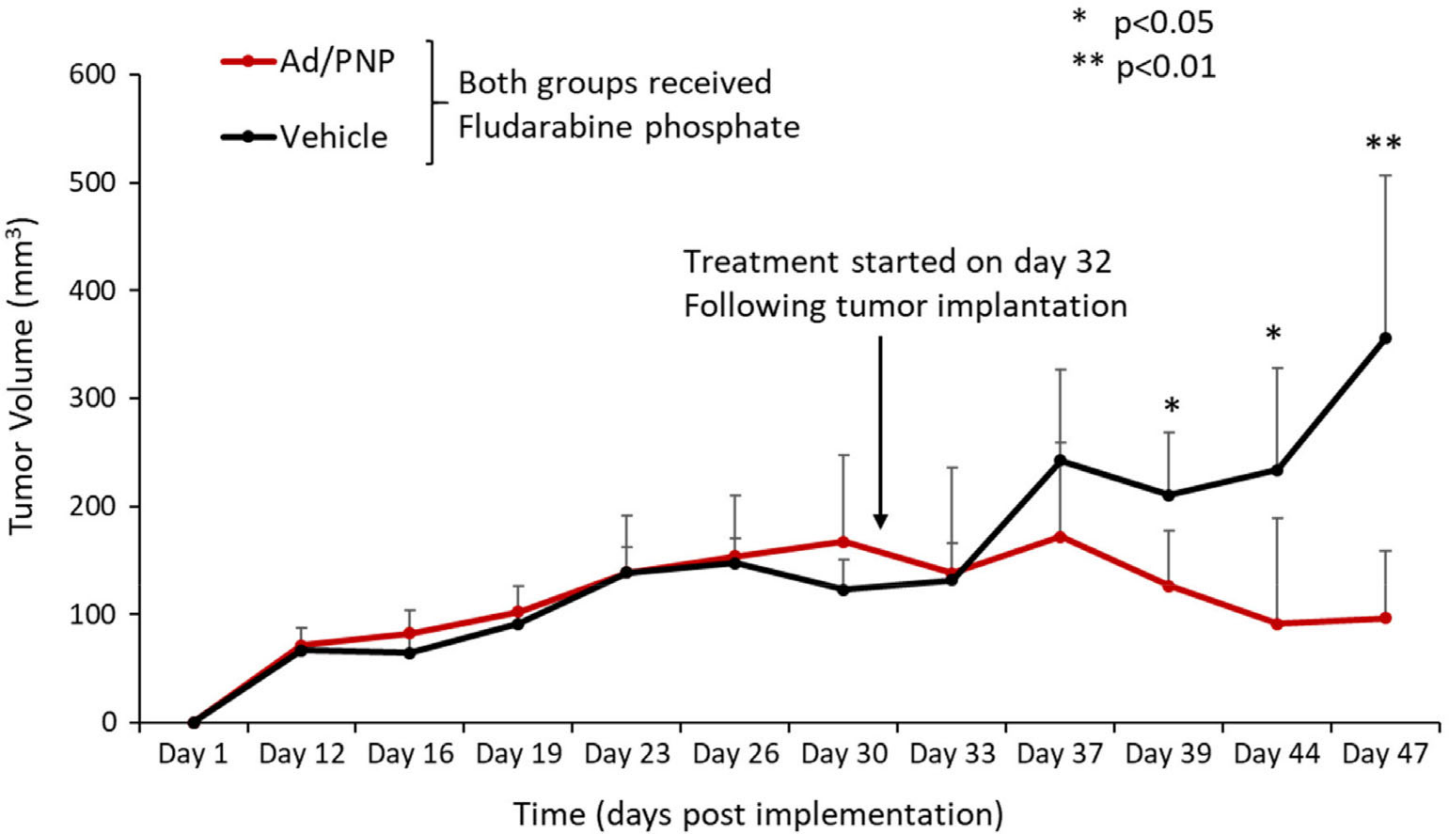
Antitumor activity in vivo. Nude mice were injected with 1 × 10^7^ PDX (328373‐195‐R‐J1‐PDC) cells in each flank. When tumor sizes reached approximately 100–300 mm^3^, animals were divided into two experimental groups in which tumors were treated with intralesional Ad5‐PNP (2 × 10^11^ viral particles in 150 μl three times a day at 4‐h intervals × 2 days) or vehicle. Both cohorts of mice were given F‐araAMP (167 mg/kg intraperitoneally three times per day × 3 days). *N* = 6–7 tumors per group. Bars show standard error of mean. Mean tumor volumes are shown (*p < .05; **p < .01)

Based on studies described above and earlier reports that intratumoral PNP leads to robust activity against non‐head and neck cancer lines,^3^, ^5^, ^6^, ^7^, ^8^ we examined PDX‐derived HNSCC following treatment with F‐araAMP alone or in combination with Ad5‐PNP. Schedule and timing of Ad5‐PNP and F‐ara‐AMP were determined from previous data showing: (1) transgene expression following Ad5‐PNP is maintained in solid tumors after 4–5 days, and (2) fludarabine phosphate at 167 mg/kg is safe and effective in other tumor settings^3^ (and unpublished observations). PDX tumors given Ad5‐PNP + F‐araAMP exhibited decreased growth compared to mock infected neoplasms receiving a single treatment cycle of F‐araAMP (Figure 3). Experiments such as these provide a foundation for augmenting PNP therapy in the future, including multiple (repeat) cycles of fludarabine to enhance efficacy, changes in the volume or amount of virus, hydrostatic pressure during intralesional inoculation, duration or schedule of needle injection, and so forth.

### 3.5 Adenovirus receptor expression in PDX cells

**FIGURE 4:**
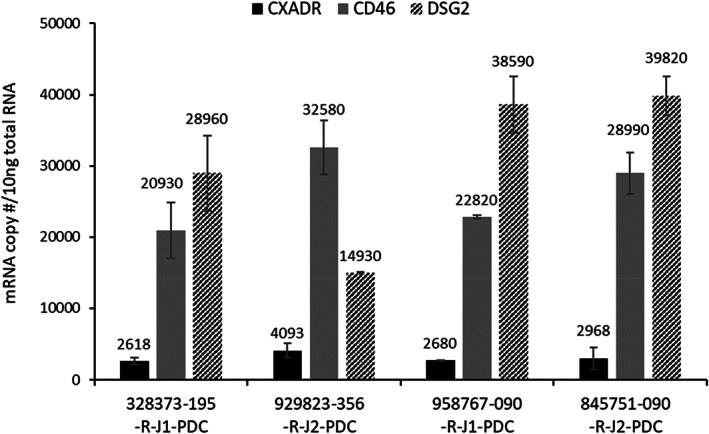
Adenovirus receptor expression in head and neck tumors. Levels of specific mRNAs in 10 ng total RNA for each human PDX model are shown. (CXADR: Ad5 receptor CAR; CD46 and DSG2: Ad3 receptors CD46 and desmoglein 2). *p* values calculated by student's *t* test. Levels of mRNA between the four cell models varied by up to 1.6‐fold (CXADR), 1.6‐fold (CD46), or 2.7‐fold (DSG2)

Adenovirus 5 (subgroup C) enters cells via CAR^26^ (i.e., CXADR; Coxsackievirus and adenovirus receptor). Suominen et al.^27^ reported CAR expression was comparatively low in primary HNSCC, a finding that could limit Ad5 transduction. In contrast, CD46 expression is abundant in HNSCC tissues (Figure 4), and retargeting of adenovirus to CD46 has been reported as efficient.^28^ CD46 and DSG2 each serve as high affinity cell surface receptors for Ad3 (subgroup B),^29^, ^30^ and studies to test receptor abundance in PDX human tumor models could be of considerable value. Accordingly, we investigated levels of adenoviral receptor proteins in PDX tumors, and observed CD46 and DSG2 expression at much higher levels than CAR (Figure 4), indicating potential usefulness as superior targets for viral gene delivery.

### 3.6 Ad5‐F5/3 transduces PDX cells and confers increased levels of therapeutic transgene

**FIGURE 5:**
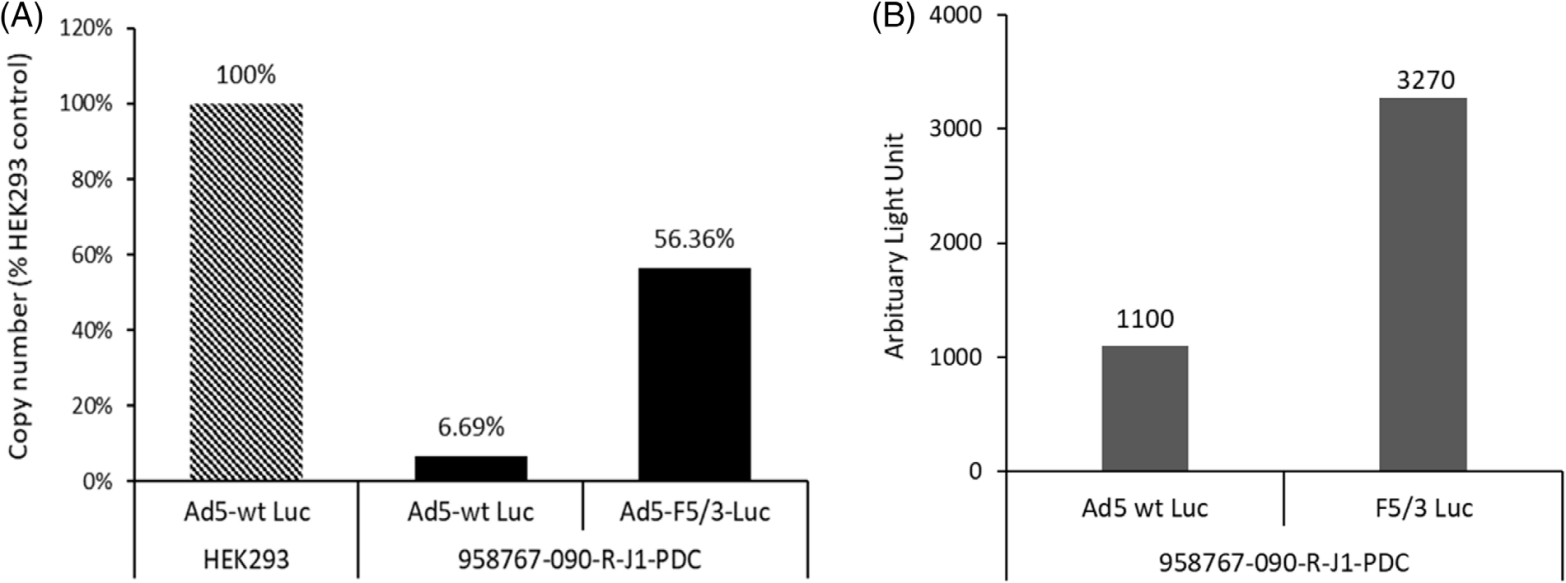
Viral infectivity and transgene expression. Ad5‐luc and Ad5‐F5/3‐luc entry into HEK293 or human head and neck PDX cancer models were evaluated by measuring intracellular adenoviral genomic DNA based on copy number variance ddPCR. In each experiment, 4 × 10^12^ viral particles were added to tumor cells and genomic DNA (viral and cellular) prepared with copy number variance determined on a QX200 ddPCR system. Luciferase expression levels from Ad5‐luc or Ad5‐F5/3‐luc were also evaluated (at 48 h following infection). (A) Infectivity (quantified as intracellular viral genome count) shown as % of Ad5‐wt Luc in HEK293 cells. (B) Luciferase expression (1 × 10^5^ cells infected with 4 × 10^8^ viral particles)

As an example of ways altered viral tropism might be evaluated and enhanced using leading‐edge PDX models, we performed a study with type 5 adenovirus (Ad5) containing a chimeric capsid protein fiber engineered to incorporate the C‐terminal knob (receptor binding) domain of adenovirus type 3 (subgroup B). The construct was evaluated for improved HNSCC transduction and reporter gene expression (Figure 5). Intracellular viral genome count using ddPCR showed substantially increased gene transfer by Ad5‐F5/3 compared with non‐modified (first generation) Ad5 (Figure 5A). Improved reporter gene (luciferase) signal was also observed using Ad5‐F5/3 (Figure 5B, see also below).

## 4 DISCUSSION

*E. coli* PNP‐based nucleoside cleavage represents an emerging experimental therapy for HNSCC, and optimizing this strategy depends on availability of well validated preclinical model systems.^3^, ^5^, ^6^, ^7^, ^8^, ^9^, ^10^, ^11^, ^12^, ^13^, ^14^, ^15^, ^16^, ^17^, ^18^, ^19^ Little is known regarding uptake mechanisms for recombinant adenovirus in head and neck tumors, limits to cell killing by MeP or F‐Ade, relationship(s) between HNSCC tumor size and PNP‐based efficacy, etc. Moreover, radiation and other modalities^3^, ^4^ have been shown to improve PNP‐dependent regression of glioma tumors, whereas robust HNSCC models for studies of this sort are not widely available. Our findings provide a new foundation for optimizing preclinical treatment of head and neck cancer—with relevance to PNP clinical protocols currently in progress.

Human HNSCC tumors that faithfully recapitulate aspects of viral vector uptake, uncoating, and expression of therapeutic transgenes were evaluated in the present experiments. We examined chemotherapeutic susceptibility (Figure 1), adenoviral gene transfer (Figure 2), in vivo tumor regression following viral transduction (Figure 3), tumor expression profiling of viral receptors (Figure 4), and modified viral tropism (Figure 5) – all of which address significant gaps in knowledge regarding HNSCC. Although data from other (non‐HNSCC) tumor types were sufficient to gain FDA investigational new drug approval and clinical testing of the PNP technology, additional preclinical information would be valuable regarding this specific strategy against HNSCC. The NCI repository provides tumors from distinct donors, and allows spectrum of activity and fundamental mechanism to be addressed using malignant tissue from numerous head and neck cancer patients.

F‐Ade is a purine molecule that exhibits robust killing of cancer cells in vitro and in vivo. The precursor compound, F‐araAMP is rapidly de‐phosphorylated in the circulation to generate a bioavailable molecule (F‐araA) that serves as an *E. coli* PNP substrate. F‐Ade is released by the prokaryotic PNP (but not the mammalian enzyme),^31^, ^32^, ^33^, ^34^ and subsequently metabolized by adenine phosphoribosyltransferase (APRT)^33^, ^35^, ^36^, ^37^ to cytotoxic compounds which become incorporated into cellular DNA and RNA, and also inhibit protein synthesis.^3^

Previous in vitro and animal studies have shown tumor ablation using the PNP approach, including analysis in murine models of human and murine breast, primary lymphoma, glioma, hepatoma, pancreatic, prostate, and other cancers^3^, ^7^, ^10^, ^11^, ^12^, ^13^, ^14^, ^15^, ^16^, ^17^, ^18^, ^19^, ^38^, ^39^ (and unpublished observations). Our results in the current study using human PDX HNSCC models further establish broad spectrum of activity for PNP as an anticancer intervention. Moreover, use of patient‐derived HNSCC models are expected to more faithfully reproduce features of tumors in human subjects compared with immortalized cell lines. High‐level anticancer activity of purine base molecules, rapid tumor cell killing, safety of intratumoral chemotherapy generation, and strong “bystander” effect represent novel and powerful aspects of the technology. To our knowledge, for example, no other gene‐directed prodrug activation strategy mediates bystander killing of the magnitude demonstrated previously for *E. coli* PNP.^2^, ^3^, ^4^, ^7^, ^8^ The robust mechanism of cell disruption mediated by PNP/fludarabine should also be active against both HPV+ and HPV‐ tumor masses, an important clinical consideration with regard to head and neck malignancy.

Establishment of an Ad5‐F5/3 chimeric virus that improves HNSCC transduction provides a novel experimental tool, as well as “proof of concept” for ways HNSCC PDX lines might be of particular value. In unpublished experiments, for example, we tested fiber modifications that enhance RGD binding (to allow integrin dependent vector uptake) and pk7 (using C‐terminal extension of the fiber knob by heptalysine for promoting endogenous heparan sulfate mediated vector internalization), but did not observe enhanced transgene delivery to PDX lines (compared to Ad5 control). Data in Figures 4 and 5 support F5/3 as a starting point for improved PNP expression (increasing the percentage of PNP transduced cells or augmenting PNP enzymatic levels on a per‐cell basis) using methods such as those employed here (Figure 3). This could be accomplished, for example, by incorporating either the *E. coli* PNP or a more active fludarabine cleavage enzyme (such as a recently described *T. vaginalis* PNP^1^) to promote efficacy. For studies such as these, PDX HNSCC models are expected to maintain native viral uptake pathways more reliably than passaged cell lines. Our findings therefore provide an approach by which PNP treatment of locally invasive head and neck malignancy can be better understood and augmented.

*E. coli* PNP has been preliminarily evaluated in human subjects with otherwise untreatable HNSCC. Ad5‐PNP vectors similar to those used in the present experiments were administered intratumorally to patients, and F‐ara‐AMP dosed intravenously.^5^ Four of six subjects with solid tumors at higher dosing levels demonstrated partial or complete response after a single treatment cycle with follow‐up to ~60 days. These early clinical findings therefore indicate promise, but also emphasize the importance of models using human tumor xenografts to help optimize transgene delivery, intratumoral PNP distribution/expression, and endpoints that predict tumor regression in vivo. The current report provides a scientific foundation for future studies of that type.

## AUTHOR CONTRIBUTIONS

**Regina Rab:** Conceptualization (equal); data curation (equal); formal analysis (equal); investigation (equal); methodology (equal); validation (equal); visualization (equal); writing – review and editing (equal). **Annette Ehrhardt:** Conceptualization (equal); data curation (equal); investigation (equal); methodology (equal); supervision (equal); visualization (equal); writing – review and editing (equal). **Bhagelu R. Achyut:** Data curation (equal); formal analysis (equal); investigation (equal); visualization (equal); writing – review and editing (equal). **Disha Joshi:** Data curation (equal); investigation (equal); methodology (equal); validation (equal); writing – review and editing (equal). **Melissa Gilbert‐Ross:** Funding acquisition (equal); methodology (equal); project administration (equal); resources (equal); supervision (equal); writing – review and editing (equal). **Chunzi Huang:** Data curation (equal); investigation (equal); methodology (equal). **Katharine Floyd:** Data curation (equal); investigation (equal); methodology (equal). **Anton V. Borovjagin:** Funding acquisition (equal); investigation (equal); methodology (equal); resources (equal); writing – review and editing (equal). **William B. Parker:** Conceptualization (equal); data curation (equal); formal analysis (equal); funding acquisition (equal); investigation (equal); methodology (equal); project administration (equal); supervision (equal); writing – review and editing (equal). **Eric J. Sorscher:** Conceptualization (equal); data curation (equal); formal analysis (equal); funding acquisition (equal); investigation (equal); methodology (equal); project administration (equal); resources (equal); supervision (equal); validation (equal); visualization (equal); writing – original draft (equal); writing – review and editing (equal). **Jeong S. Hong:** Conceptualization (equal); data curation (equal); formal analysis (equal); investigation (equal); supervision (equal); validation (equal); visualization (equal); writing – review and editing (equal).

## CONFLICT OF INTEREST

Parker and Sorscher have ownership interests in PNP Therapeutics and serve on the Board of Directors for the company, which develops products used in research described by the paper. Parker and Sorscher are also inventors of technology being evaluated in studies described by this report. The terms of this arrangement for Sorscher have been reviewed and approved by Emory University in accordance with its conflict‐of‐interest policies. Jeong Hong also has minor equity interest in this company. Other authors have no conflict‐of‐interest to report.

## ETHICS STATEMENT

All protocols complied with Emory University Institutional Animal Care and Use guidelines.

## Data Availability

The data that support the findings of this study are available from the corresponding author upon reasonable request.
